# Dynamic changes in transcripts during regeneration of the secondary vascular system in *Populus tomentosa *Carr. revealed by cDNA microarrays

**DOI:** 10.1186/1471-2164-10-215

**Published:** 2009-05-11

**Authors:** Minjie Wang, Xiaoli Qi, Shutang Zhao, Shougong Zhang, Meng-Zhu Lu

**Affiliations:** 1 Laboratory of Biotechnology, Research Institute of Forestry, Chinese Academy of Forestry, Beijing 100091, PR China; 2 College of Life Science, Northeast Forestry University, Harbin 150040, PR China

## Abstract

**Background:**

Wood is the end product of secondary vascular system development, which begins from the cambium. The wood formation process includes four major stages: cell expansion, secondary wall biosynthesis, lignification, and programmed cell death. Transcriptional profiling is a rapid way to screen for genes involved in these stages and their transitions, providing the basis for understanding the molecular mechanisms that control this process.

**Results:**

In this study, cDNA microarrays were prepared from a subtracted cDNA library (cambium zone *versus *leaf) of Chinese white poplar (*Populus tomentosa *Carr.) and employed to analyze the transcriptional profiles during the regeneration of the secondary vascular system, a platform established in our previous study. Two hundred and seven genes showed transcript-level differences at the different regeneration stages. Dramatic transcriptional changes were observed at cambium initiation, cambium formation and differentiation, and xylem development, suggesting that these up- or downregulated genes play important roles in these stage transitions. Transcription factors such as AUX/IAA and PINHEAD, which were previously shown to be involved in meristem and vascular tissue differentiation, were strongly transcribed at the stages when cambial cells were initiated and underwent differentiation, whereas genes encoding MYB proteins and several small heat shock proteins were strongly transcribed at the stage when xylem development begins.

**Conclusion:**

Employing this method, we observed dynamic changes in gene transcript levels at the key stages, including cambium initiation, cambium formation and differentiation, and xylem development, suggesting that these up- or downregulated genes are strongly involved in these stage transitions. Further studies of these genes could help elucidate their roles in wood formation.

## Background

Wood constitutes one of the most important natural resources on earth, and is a potential future alternative to fossil energy resources. Wood is the end product of secondary vascular system (SVS) development, and is mainly composed of fibers and tracheary elements with secondary cell walls [1]. Four major processes are involved in wood formation: cell expansion, secondary wall biosynthesis, lignification, and programmed cell death. Recent advances in the understanding of these processes have revealed that wood formation is under highly regulated genetic control [1]. In recent years, genomic approaches have become the primary techniques for exploring wood formation. Large EST databases have been generated from different tissues of poplar [2-4], black locust [5], and loblolly pine [6]. Meanwhile, transcriptional profiles have been revealed using cDNA microarrays of wood-forming tissues of pine [7,8], black locust [5], eucalyptus [9], and poplar [10-12]. These studies have demonstrated that hundreds of genes are involved in wood formation.

Most previous work in this area has investigated genes that are differentially expressed between stems and other organs or among different wood-forming tissues, lacking information on the changes in gene expression that occur during different stages of wood formation. In particular, genes expressed only at transitions between different stages are likely to control the entire process of wood formation. The regeneration of the poplar SVS, developed in our laboratory [13], which mimics the initiation and differentiation of cambium cells into vascular systems, provides an opportunity to follow key stages of wood formation by sampling clonal trees at different regeneration stages. In this work, cDNA microarrays derived from a subtractive library of cDNAs of cambium zone *versus *leaf were used to analyze the genes that show different transcript levels during the different stages of SVS regeneration. Our results show that this approach provides an efficient way to identify dynamically expressed genes that are likely to be involved in the control of SVS development in poplar.

## Results

### Regeneration of the SVS in *P. tomentosa*

The regeneration processes observed were similar to those described in our previous work [13]. Briefly, cambial zone was peeled off with the bark, leaving a few layers of immature xylem on the surface of the girdled trunk. At 2 days after girdling (AG), the immature xylem ray cells near the surface had divided, forming callus. Discontinuous meristem cells were observed at 6 days AG at the remaining immature xylem, and at 10 days AG, nearly continuous, flat meristem cells in an irregular arrangement (separated by a few parenchyma cells) were observed. At 14 days AG, the flat meristem cell layer had increased in size and formed a vascular cambium with a continuous and regular layer, consisting of fusiform initials and ray initials. The newly formed cambium was observed to be differentiating at 18 days AG. Large numbers of xylem cells and phloem cells derived from the cambium zone were observed at 22 days AG, and a normal wood-forming structure had completely regenerated. Additional file 1 shows the process of SVS regeneration.

### Construction of a cambium zone-specific cDNA subtractive library and production of cDNA microarrays

To generate a cDNA library enriched in cambial zone-specific genes, the SSH technique was chosen because it equalizes the representation of both rare and abundant transcripts. cDNAs were prepared using total RNAs isolated from pools of cambium tissue and immature leaves of three trees in a clonal plantation to minimize possible individual variations. More than 2,400 positive clones were obtained from the subtractive library, and 1,197 clones with inserts of at least 100 bp in length were selected after amplification of the inserts. The 1,197 fragments were PCR-amplified and spotted onto poly-L-lysine coated slides to produce cDNA microarrays.

### cDNA microarray analysis of transcriptional profiles during SVS regeneration

**Figure 1:**
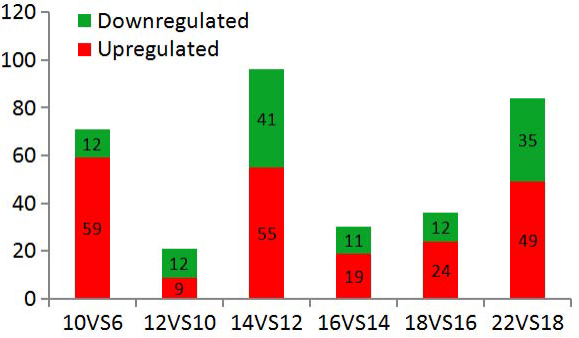
**Number of genes with differences in transcript levels between adjacent stages of SVS regeneration**. The numbers of genes showing differences in transcript levels between adjacent stages (*x*-axis) during SVS regeneration as determined by cDNA microarray analysis are shown; 363 genes were shown to have dynamic transcript levels during SVS regeneration. Larger numbers of genes were up- or downregulated on days 10, 14, and 22 AG than at other points, indicating that the transcriptional profiles changed dramatically at these key switches between regeneration stages.

Probes prepared from different regeneration stages, at 6 days (meristem cell initiation), 10 days (cambial tissue initiation), 12 and 14 days (cambium formation), 16 and 18 days (cambial cell differentiation), and 22 days (xylem formation) AG, were hybridized to the cDNA microarrays. The transcript levels of different genes (assuming one cDNA fragment represents one gene) during the adjacent regeneration stages were compared, and 338 genes displayed different transcript levels (Fig. 1). In total, 207 genes were found to be up- or downregulated during SVS regeneration (Additional file 2) after redundant genes (in which differences in transcript levels were observed during more than one regenerating stage) were removed. Larger numbers of genes were up- or downregulated on days 10, 14, and 22 AG than at other points, indicating that the transcript levels changed dramatically at these key switches between regeneration stages (Fig. 1).

**Figure 2:**
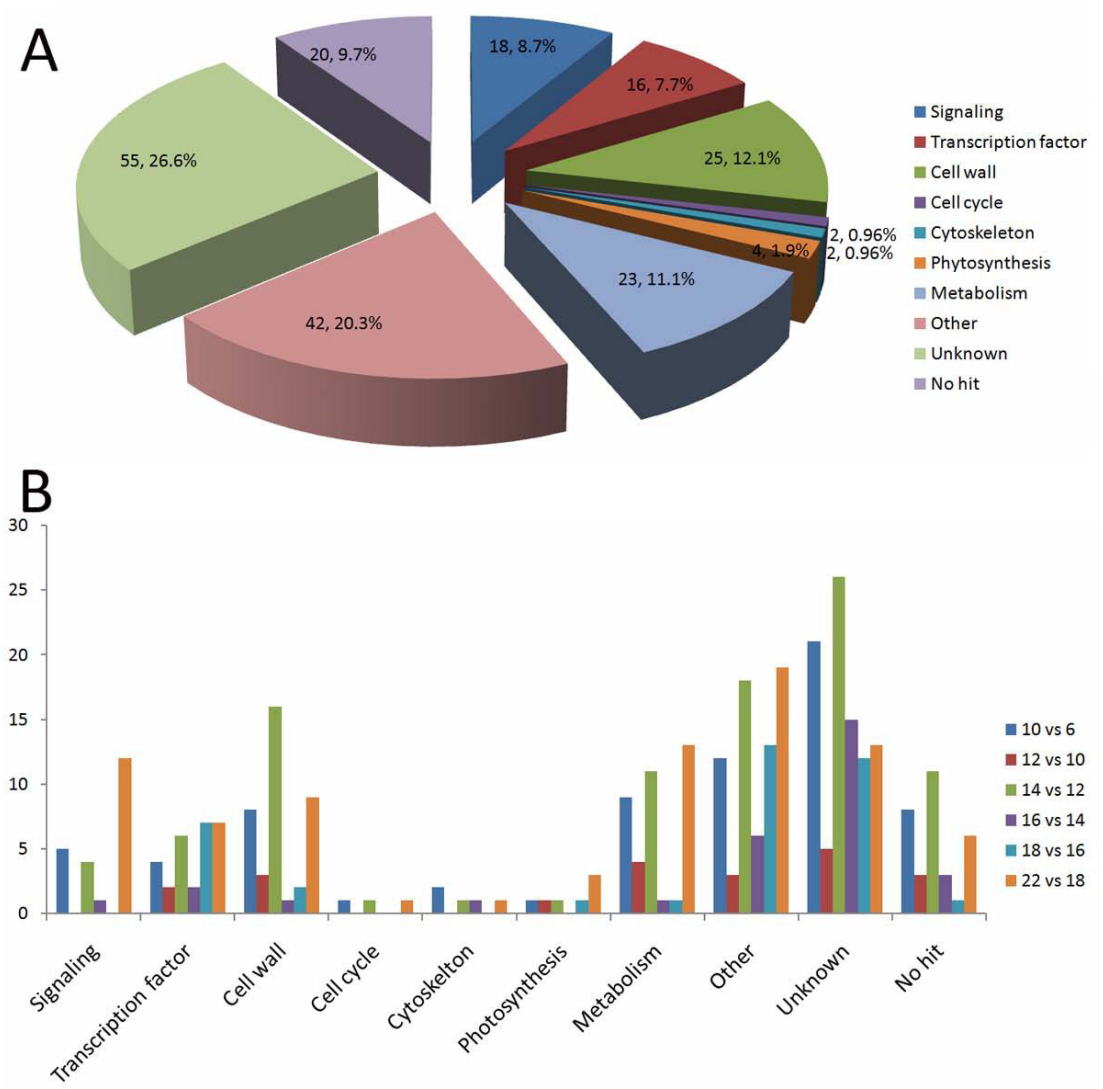
**Functional classification of genes with transcriptional dynamics during SVS regeneration**. The genes were categorized as those encoding transcription factors; those related to signaling and stress, cell wall synthesis, cell cycle regulation and cell division, the cytoskeleton, photosynthesis, or metabolism; those having other functions; and as "unknown" or "no hits". A: The proportion of each data set is displayed. B: The number of identified genes in each category for each regeneration stage is shown.

The above 207 genes were functionally classified into nine groups (Fig. 2A), including transcription factors (7.7%), genes related to signaling and stress (8.7%), cell wall synthesis (12.1%), cell cycle regulation and cell division (0.96%), cytoskeleton (0.96%), photosynthesis (1.9%), metabolism (11.1%), other functions (20.3%), and genes whose functions are unknown (26.6%) or that produced no hits (9.7%). The transcriptional patterns of these genes during the different regeneration stages are outlined in Fig. 2B.

At the key switches of SVS regeneration (at 10, 14, 18, and 22 days AG), the expression of genes encoding transcription factors, signaling, cell wall-associated proteins, and proteins with unknown functions exhibited highly dynamic changes (Fig. 2B), in accordance with the key regeneration stages of the initiation, formation, and differentiation of vascular cambium and xylem development (See additional file 1). Interestingly, a large number of the genes (9.7%) classified as "no hits" showed altered transcript levels during SVS regeneration, suggesting that these genes are only present in trees and might play important roles in wood formation. Additional file 3 summarizes the selected genes with changed transcript levels and their dynamic transcriptional profiles during SVS regeneration that are discussed in this paper, including genes encoding transcription factors, signaling pathway molecules, enzymes involved in metabolism or cell wall biosynthesis, and "unknown" factors.

Compared to previous results [10] of transcriptional profiles of wood formation from different SVS tissues, 95 (45.9%) of the 207 genes obtained in our study shared the same accession number in *Arabidopsis *as described in the previous study, and 44 (21.3%) were identical or similar to a gene description of the previous study. However, the other 68 (32.9%, additional file 2) were not shared by the two studies, and had neither the same accession number nor the same gene description.

We compared the 132 putative genes from microarray data with the 199 proteins previously obtained during the SVS regeneration using a proteomic approach [13], and 35 (27%, additional file 2) of the genes shared the same or similar function with the counterparts. Notably, genes encoding transcription regulators (calmodulin-related, auxin-induced protein, nodulin-like protein, thaumatin-like protein, NIMA-related protein kinase, receptor protein kinase, protein-tyrosine kinase HTK), transcription factors (4 MYB-family proteins), heat shock proteins (3), and lignin biosynthesis enzymes (cinnamate 4-hydroxylase and 2 4-coumarate:CoA ligase) were found in both studies, and the involvement of some of these genes in the regeneration of SVS is discussed further (see Discussion). The relatively low number of genes shared by these two studies could have been due to the limitation regarding the total number of genes found by these two approaches, as well as differences in gene expression at the transcriptional and translational levels.

### Validation of cDNA microarray data by real-time PCR

**Figure 3:**
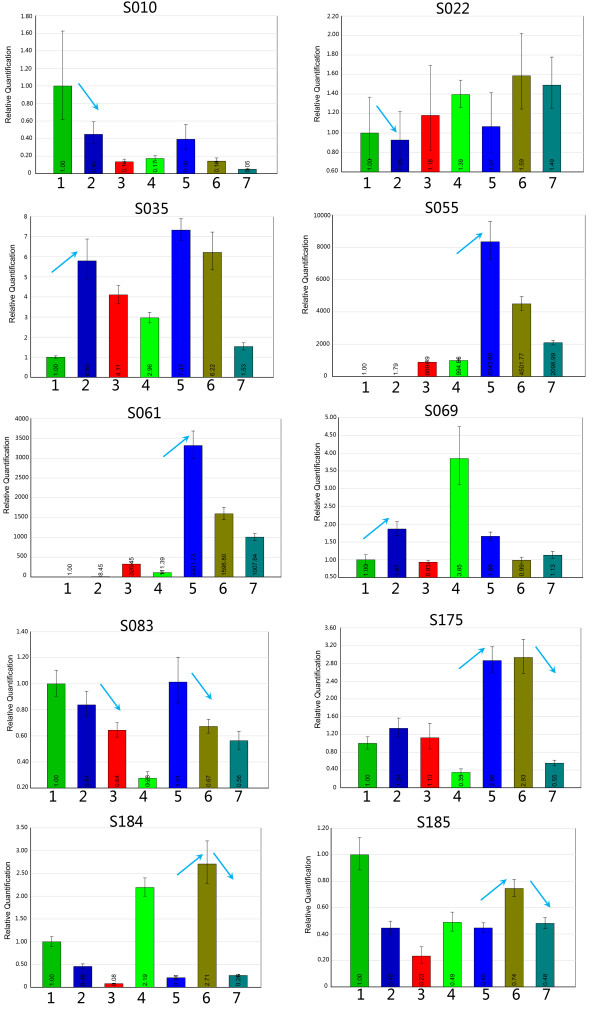
**Validation of dynamic transcriptional profiles during SVS regeneration by real-time PCR**. Light-blue arrows indicate the transcriptional trend of the same gene between adjacent stages as revealed by microarray analysis. The *x*-axis shows the regeneration stages at 6, 10, 12, 14, 16, 18, and 22 days AG; the *y*-axis shows the ratio of the calibrated transcript accumulation data of each stage to that at 6 days AG.

The cDNA microarray analysis provided a global view of the dynamic transcriptome of wood formation. To further verify the data, the transcript levels of ten genes (S010, S022, S035, S055, S61, S69, S83, S175, S184, and S185 in additional file 3) at different regeneration stages were analyzed by real-time PCR (Fig. 3). Similar transcription trends were obtained using the two methods, although the extent of accordance for the transcript levels of a given gene varied. For instance, the microarray data showed that the gene S175 was strongly transcribed at the stage of differentiation of the cambium into the xylem (Additional file 3), and the same transcriptional pattern was obtained by real-time PCR. The similar trends of the transcriptional dynamics obtained by both cDNA microarray analysis and real-time PCR suggest that the transcriptional profiles of these genes during regeneration are generally reflected by the microarray data.

## Discussion

To identify essential genes that play important roles in wood formation, an efficient approach is to characterize genes that are expressed at key switches of SVS development. We used cDNA microarrays and our previously established SVS regeneration system to identify genes that are preferentially expressed at the key switches of the SVS regeneration process. Similar to the results of our previous proteomics study [13], this work found that the transcript level also changed dramatically at these key switch points, at which the anatomy, structures, and functions of the cells change with the appearance of new types of cells and tissues. More than 67% of the genes we identified had the same function description as in a previous work by Hertzberg and coworkers [10], verifying the results and further confirming their involvement in wood formation. However, a large number of the genes (9.7%) were only identified in our study. This could have occurred because the previous study [10] only analyzed the transcriptional profiles in the cambium zone and immature and mature xylem, whereas we analyzed the transcriptional profiles following the dynamic process of SVS regeneration, allowing us to obtain genes with more dynamic changes in transcript levels or those that are only strongly transcribed during the transitions. For instance, the gene encoding the Ag13 protein precursor (S050) was only transcribed strongly at 18 days AG, when the differentiation of cambium into xylem was beginning, and was transcribed at low levels during the other stages.

In the early stages of SVS regeneration, meristem cells appeared at 6 days AG, possibly from dedifferentiated immature xylem cells; the cells then increased in number by cell division and finally had formed continuous cambium-like layers at 10 days AG. At 14 days AG, a vascular cambium had formed as a continuous and regular layer. During this process, several genes encoding transcription regulators (including PINHEAD and AUX/IAA), primary cell wall-associated proteins, and proteins of unknown function were up- or downregulated, indicating their functions in cell differentiation and meristem formation. PINHEAD was upregulated only at the stage of initiation of meristem cells, and was downregulated after 10 days AG (S003, additional file 3). In *Arabidopsis*, PINHEAD is expressed in the vasculature, and the development of shoot apical meristems is abnormal in the pinhead mutant. The PINHEAD gene should encode a component of a hypothetical meristem-forming competence factor and may play a role in primary meristem initiation [14,15]. We hypothesize that the strong expression of PINHEAD triggers certain cells to dedifferentiate into meristem cells at 6 days AG, and is no longer needed when the nearly continuous cambium-like layers have formed. Therefore, PINHEAD may be involved in cambium initiation rather than cambium maintenance. Recent advances have shown that several plant growth regulators, including auxin [16-18], cytokinin [19,20], brassinosteroid [21,22], and proteoglycan [23], regulate the development of SVS as signaling substances. A radial concentration gradient of auxin forms across the cambium region in the aspen stem and is related to secondary xylem development [17]. Mutations in several auxin response factors (ARFs) or auxin (IAA)-induced proteins disrupt the normal body organization along the apical-basal axis and result in discontinuous and reduced vascular formation [24,25]. An IAA-induced gene (S069, additional file 3) is expressed at 10 days AG, suggesting that the auxin-activated expression of IAAs regulates the transcription of genes involved in cambium formation.

The genes expressed in the cambium zone were revealed by a cDNA array analysis employing the microsectioning technique, and marker genes for cambial initials (Peakset 2), phloem (Peakset 1), and xylem mother cells (Peakset 3) were found, with totals of 19, 103, and 36 genes, respectively [26]. In contrast, in our study, the initiation of cambial cells, rather than maintenance of the stem layer in the normal cambial zone sampled in the previous study, could be traced, allowing us to obtain a large number (71, additional file 2) of up- and downregulated genes involved in the transition from callus cells to stem cells. In addition, considerable numbers (41%) of these genes were of unknown function, indicating they might be novel genes that play roles in this process. Nevertheless, nine genes in our data were found that had the same (6) or a similar (3) function description as the nine counterparts (47%) in Peakset 2 for cambial initials, suggesting that these genes are important for both the formation and maintenance of the stem layer; therefore, further investigation into their roles in cambial activity is warranted. Similarly, our SVS regeneration system identified genes expressed during the differentiation of stem cells (increased cell layers in the cambium zone) into these mother cells and the maintenance of their identity, and 56 such genes (42% of the total genes of known function) in our data had the same or similar function description as markers in Peaksets 1 and 3 in the previous study [26]. However, we could not partition these dynamically transcribed genes into phloem and xylem mother cells as in the previous study due to the limitation of our regeneration system in which these two types of cells appeared at nearly the same time. In summary, we studied the temporal expression of genes involved in cambial activity, whereas the previous work [26] emphasized genes that are spatially expressed across the cambium zone. In addition, both studies found common genes of interest that play roles in this process. Combining these two experimental systems to comprehensively profile the genes that are spatially and temporally involved in SVS development by employing whole-genome poplar chips should provide valuable results.

In the later stages of SVS regeneration (from 16 to 22 days AG), the newly formed cambium zone began to differentiate, and finally a normal wood-forming structure was completely regenerated. Genes encoding MYB-family proteins (S002, S111, S112, S129, additional file 3) and several small heat shock proteins (HSPs) (S174, S184, S188, additional file 3) were either up- or downregulated. A promoter analysis of genes that encode enzymes associated with lignin biosynthesis revealed that AC elements are necessary for xylem-localized gene expression [27,28]. In *Eucalyptus*, EgMYB2, a member of the R2R3-MYB family, can bind to the promoters of the *EgCCR *and *EgCAD2 *genes, which contain AC elements, and regulate their transcription [28]. The thickness of the secondary cell wall of transgenic tobacco plants overexpressing *EgMYB2 *was dramatically increased and the lignin profile was altered, suggesting that EgMYB2 is a positive regulator of both lignin biosynthesis and secondary wall formation in xylem [28]. *Arabidopsis *undergoes secondary growth in roots, hypocotyls, and stems under certain conditions [29-33]. The transcriptional profiling of induced secondary growth in *Arabidopsis *showed that several MYB transcription factors regulate the activities and differentiation of xylem and phloem [34]. Our results showed MYB-family genes transcribed at high levels at 18 and 22 days AG (S002, S111, S112, S129, additional file 3), revealing that certain MYB proteins are involved in the regulation of xylem development.

Interestingly, during the regeneration process, rare genes encoding enzymes involved in lignin biosynthesis were found to differ at the transcript level, except for genes encoding 4-coumarate:CoA ligase (4CL) (S141, S155, additional file 3) and cinnamate 4-hydroxylase (C4H) (S036, additional file 3). However, the expression of genes associated with cell wall biosynthesis and assembly, such as extensins and expansins, changed dramatically, reflecting the transitions of cell types during this regeneration process but without large changes in the chemical composition of the cell walls. Since our cDNA microarray was prepared from a cambium zone-specific subtractive library, and the regenerated tissues mainly included cambium, immature xylem, and a small amount of phloem, lacking secondary cell wall deposition, the expression of most of the genes associated with lignin biosynthesis was not extensively observed.

Small HSPs, induced by gibberellins or methyl jasmonate, play roles in plant growth and development [35], including wood formation [36]. The transcripts of several small HSPs (S174, S184, S188, additional file 3) changed dramatically at 16, 18, and 22 days AG in our study, indicating that small HSPs are highly involved in cambium differentiation and xylem development. In addition, several genes of unknown function were strongly transcribed at key switches of the regeneration process, suggesting their potential roles in SVS regeneration. The transcript levels of genes encoding the P0510C12.9 protein at 10 days AG were 12- to 37-fold higher than at 6 days AG, and the high transcript levels persisted through the regeneration process. At 16 days AG, the transcription of the P0510C12.9 gene (five such genes were found, but only S061 is listed in additional file 3) was significantly stronger than at 14 days AG. These proteins of unknown function might be important players in wood formation. The transcription of numerous genes encoding these HSPs and unknown proteins was found to be highly dynamic during this process, and it would be interesting to further elucidate their roles in wood formation.

## Conclusion

To understand the molecular mechanisms that occur during the development of the SVS, further efforts should be focused on characterizing the functions of these strongly and dynamically transcribed genes to investigate their roles in wood formation. These studies should include the identification of the target genes of pivotal transcription factors, demonstration of the aforementioned hierarchical network of transcriptional regulation, elucidation of the modes of interaction between signaling molecules and transcription factors, and exploitation of different types of data related to posttranscriptional, translational, and posttranslational genetic control.

## Methods

### Plant materials and tissue harvesting

Healthy *P. tomentosa *individuals in a 4-year-old clonal plantation (located in Renqiu, Hebei Province, China) were debarked and treated as described previously [13]. The cambial zone, including soft tissue (immature xylem) on the debarked trunk and the inner side of the peeled bark, was collected to construct a subtractive cDNA library. Samples were subsequently collected by scraping regenerated tissues from the surfaces of trunks at 6, 10, 12, 14, 16, 18, and 22 days AG, and immediately frozen and stored in liquid nitrogen. Sampling was performed on three clonal trees at one time, and the samples from each individual tree were pooled before RNA extraction.

### Total RNA extraction, construction of a cDNA subtractive library, and production of cDNA arrays

Total RNAs were extracted from 100 mg each of cambial zone (a small amount of phloem and immature xylem tissues were also included) and immature leaf using the RNeasy Plant Kit (Qiagen) with DNase treatment. The cambium zone (tester) versus immature leaf (driver) subtractive library was constructed using the suppression subtractive hybridization (SSH) technique [37]. SSH was performed with the PCR-Select cDNA Subtraction Kit (Clontech) according to the manufacturer's instructions. The subtracted PCR products generated by SSH were inserted into the pGEM-T Easy vector (Promega) and transformed into *Escherichia coli *strain DH5α. Positive clones with an insert longer than 100 bp were isolated, and the inserts were amplified by PCR using the vector T7 and SP6 sequences as primers. PCR products longer than 100 bp were adjusted to 0.8–1.0 μg/μl, denatured by adding an equal volume of DMSO, and spotted onto poly-L-lysine-coated slides (Sigma) using a GeneMachines OmniGrid™ 100 microarrayer (Genomic Solutions). cDNA fragments and total DNA from *P. tomentosa *were arranged in triplicate in 18 × 17 spot arrays.

### Probe labeling, hybridization, image acquisition, data processing, and statistical analysis

Total RNAs were extracted from 100 mg of samples harvested on different days AG using the RNeasy Plant Kit (Qiagen) with DNase treatment. Double-stranded cDNA was synthesized from 10 μg of total RNA using a cDNA Synthesis Kit (TaKaRa), and then transcribed into cRNA *in vitro *using the T7 RiboMAX Express Large Scale RNA Production System (Promega). Two micrograms of cRNA were reverse-transcribed by Superscript II (Invitrogen), and 1-μg aliquots of products were labeled with Cy5 or Cy3 using the DNA polymerase Klenow fragment. The labeled DNA was purified and dissolved in 30 μl of hybridization buffer (3× SSC, 0.2% SDS, 5× Denhardt's solution, 25% formamide). Hybridization was carried out at 42°C for 14–16 hours. The slides then were washed once at 42°C in 2× SSC + 0.2% SDS for 5 minutes, and once at room temperature in 0.2× SSC. The slides were scanned using a GenePix 4000B Array Scanner (Axon Instruments). Two samples of adjacent stages were hybridized against each other four times, including dye swaps. For statistical analysis, parametric *t*-tests were performed on the normalized microarray data using GenePix Pro 4.0 (Axon Instruments) and the data were submitted to ArrayExpress. Differentially transcribed genes were defined as those showing a twofold difference in transcript accumulation between two adjacent stages.

To deduce the functions of the genes found to differ in transcript levels during SVS regeneration, the corresponding clones were sequenced and the resulting ESTs were submitted to GenBank (Additional file 2). Comparative sequence analysis was conducted with the BLAST algorithm against the *Populus *database  and the *Populus *genome ver. 1.1  with the default parameters. cDNAs without any significant match were designated as having no hits.

### Real-time reverse transcription PCR

Real-time PCR was performed using a 7500 Real Time PCR System (Applied Biosystems) and a SYBR^® ^Premix Ex Taq™ Kit (TaKaRa). Primer pairs were designed using Primer Express ver. 3.0 (Applied Biosystems) to amplify fragments of between 150 and 200 bp. Total RNAs were prepared from 100 mg of samples harvested at different regeneration stages as described previously. Single-stranded cDNAs were synthesized from 2 μg of total RNA using the SMART cDNA Synthesis Kit (Clontech) and diluted 50-fold with RNase-free water. PCR reactions were prepared in 25-μl volumes containing 1 μl of diluted single-stranded cDNA, 1× SYBR^® ^Premix Ex TaqTM SYBR Green I Master Mix, and 0.5 μM of each primer (sequences of the primers used for real-time PCR are provided in the additional file 4). Six replicates were carried out in parallel, and *Ubiquitin *(BU879229) served as the endogenous control [38]. Statistical analysis of the data was performed using the SDS 1.3 installed in the 7500 Real Time PCR System.

## Authors' contributions

WMJ initiated the study, prepared the cDNA microarray, performed the array analysis, participated in the data analysis and interpretation, and drafted the manuscript. QXL carried out the real-time PCR. ZST carried out the sectioning the regenerated tissues. ZSG contributed to the experimental design. LMZ contributed to the study design, coordinated the data analysis and interpretation, and helped draft the manuscript. All authors participated in the regeneration process and sampling, and contributed to the final version of the manuscript.

## Supplementary Material

Additional file 1 **Cross sections of a portion of poplar trunks during regeneration**. Cross sections of a portion of poplar trunks during regeneration. (A) At 6 days AG, massive callus formed on the surface of a girdled trunk (ca), and discontinuous meristem cells (m) appeared; (B) at 10 days AG, showing meristem cells (m) with a flat shape inside the callus (ca); (C) at 12 days AG, the cambium-like zone (c) has formed; (D) at 14 days AG, continued cambium-like zone (c) formation between the callus and immature xylem (imx); the imx vessels remained thin in their wells; (E) at 16 days AG, enlarged cells (ec) are present inside the cambium-like zone (c); (F) at 18 days AG, the imx vessels became thickened in their wells, and a fiber cell cluster (fc) appeared; (G) at 22 days AG, the regenerated vessel (v) and phloem (ph) are shown. Bar = 100 μm. Click here for file

Additional file 2 **Genes transcribed differentially during SVS regeneration**. Additional file 2 shows 224 genes differentially transcribed during SVS regeneration. Click here for file

Additional file 3 **Selected genes that show dynamic changes in transcriptional profiles during SVS regeneration**. Additional file 3 summarizes selected genes and their dynamic changes in transcriptional profiles during SVS regeneration that are discussed in this paper, including genes encoding transcription factors, signaling pathway molecules, enzymes involved in metabolism or cell wall biosynthesis, and "unknown" factors. Click here for file

Additional file 4 **Sequences of primers used in real time RT-PCR**. Sequences of primers used in real time RT-PCR. Click here for file
