# Supplementary figures and images for: Dynamic changes in transcripts during regeneration of the secondary vascular system in Populus tomentosa Carr. revealed by cDNA microarrays
Source: BMC Genomics. 2009 May 11;10:215. doi: 10.1186/1471-2164-10-215 (PMC2685409; doi:10.1186/1471-2164-10-215)

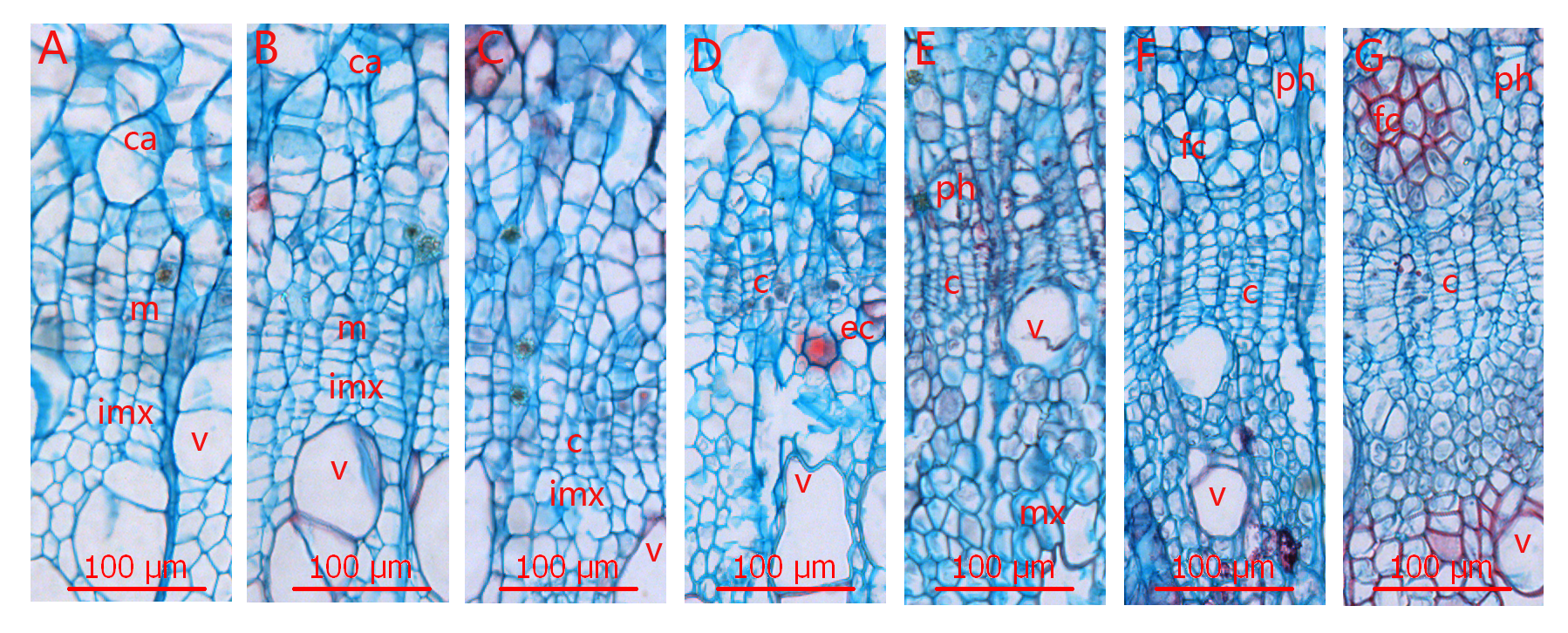

Supplement: Additional file 1 — Cross sections of a portion of poplar trunks during regeneration. Cross sections of a portion of poplar trunks during regeneration. (A) At 6 days AG, massive callus formed on the surface of a girdled trunk (ca), and discontinuous meristem cells (m) appeared; (B) at 10 days AG, showing meristem cells (m) with a flat shape inside the callus (ca); (C) at 12 days AG, the cambium-like zone (c) has formed; (D) at 14 days AG, continued cambium-like zone (c) formation between the callus and immature xylem (imx); the imx vessels remained thin in their wells; (E) at 16 days AG, enlarged cells (ec) are present inside the cambium-like zone (c); (F) at 18 days AG, the imx vessels became thickened in their wells, and a fiber cell cluster (fc) appeared; (G) at 22 days AG, the regenerated vessel (v) and phloem (ph) are shown. Bar = 100 μm. [file 1471-2164-10-215-S1.tiff]
