# Supplementary material for: Dynamic changes in transcripts during regeneration of the secondary vascular system in Populus tomentosa Carr. revealed by cDNA microarrays
Source: BMC Genomics. 2009 May 11;10:215. doi: 10.1186/1471-2164-10-215 (PMC2685409; doi:10.1186/1471-2164-10-215)
Supplement: Additional file 3 — Selected genes that show dynamic changes in transcriptional profiles during SVS regeneration. Additional file 3 summarizes selected genes and their dynamic changes in transcriptional profiles during SVS regeneration that are discussed in this paper, including genes encoding transcription factors, signaling pathway molecules, enzymes involved in metabolism or cell wall biosynthesis, and "unknown" factors. [file 1471-2164-10-215-S3.doc]

Additional file 3: Selected genes that show dynamic changes in transcriptional profiles during SVS regeneration.

| **Clone ID** | **Acc. No. (*Populus* DB)** | **Acc. No. (*Arabidopsis*)** | **Description** | **Function Classification** | **Transcriptional Pattern^*^** |
| --- | --- | --- | --- | --- | --- |
| S070 | P055D11 | At3g12390 | Nascent polypeptide-associated complex | Cell cycle | 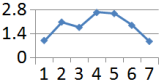 |
| S141 | POPLAR.1439.C1 | At1g20510 | 4-coumarate:CoA ligase | Cell wall | 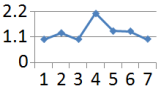 |
| S155 | FL_GENBANK_115 | At1g51680 | 4-coumarate:CoA ligase | Cell wall | 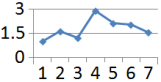 |
| S036 | UA47BPD07 | At2g30490 | Cinnamate 4-hydroxylase | Cell wall | 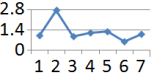 |
| S097 | S059B12 | At5g07750 | Extensin | Cell wall | 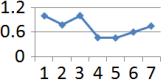 |
| S115 | S059B12 | At5g07750 | Extensin | Cell wall | 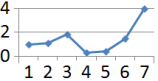 |
| S093 | R050H07 | At2g39770 | GDP-mannose pyrophosphorylase | Cell wall | 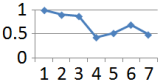 |
| S116 | V046B04 | At1g13930 | Hydroxyproline-rich glycoprotein | Cell wall | 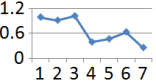 |
| S164 | UM93TD10 | At2g40370 | Laccase precursor | Cell wall | 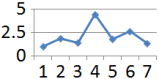 |
| S042 | A022P07 | At2g28950 | Possibly putative expansin | Cell wall | 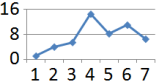 |
| S045 | A001P40.3pR | At2g28950 | Possibly putative expansin | Cell wall | 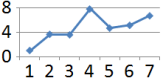 |
| S047 | G127P69.3pR | At2g28950 | Possibly putative expansin | Cell wall | 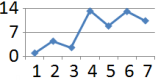 |
| S048 | A001P40.3pR | At2g28950 | Possibly putative expansin | Cell wall | 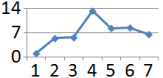 |
| S059 | A006P18 | At2g28950 | Possibly putative expansin | Cell wall | 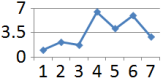 |
| S076 | UM93TD10 | At2g40370 | Possibly putative laccase | Cell wall | 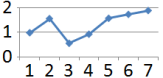 |
| S058 | UB20CPH08 | At2g28950 | Putative expansin | Cell wall | 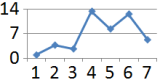 |
| S095 | UB62BPA06.3pR | At4g03210 | Xyloglucan endotransglycosylase | Cell wall | 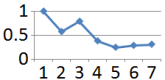 |
| S012 | T002C11 | At1g78380 | 2,4-D-inducible glutathione S-transferase | Metabolism | 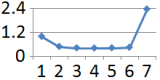 |
| S153 | A031P29.3pR | At3g48170 | Betaine aldehyde dehydrogenase-like protein | Metabolism | 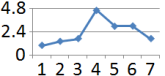 |
| **S175** | **Q032C01** | **At4g24340** | **Major storage protein** | **Other** | **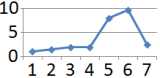** |
| **S185** | **T061A11** | **At2g47110** | **Ubiquitin extension protein** | **Other** | **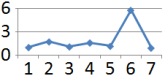** |
| S204 | Q068E07 | At1g28330 | Dormancy-associated protein | Signal | 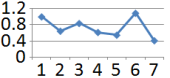 |
| S001 | S059B12 | At5g07750 | Expressed protein | Signal | 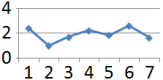 |
| S005 | G125P70.3pR | At2g38360 | Expressed protein | Signal | 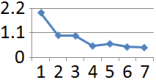 |
| S102 | A019P25.3pR | At3g15430 | Expressed protein | Signal | 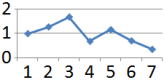 |
| S205 | UB10CPD11.3pR | At5g04690 | Expressed protein | Signal | 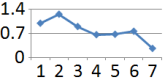 |
| S223 | B006P75.3pR | At5g15230 | Gip1-like protein | Signal | 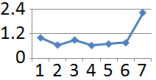 |
| S209 | B006P75.5pR | At5g15230 | Gip1-like protein. | Signal | 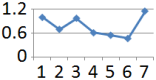 |
| S191 | I044P28 | At5g41410 | Homeotic protein BEL1 homolog | Signal | 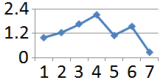 |
| S198 | UB63CPA12.3pR | At4g11260 | Hypothetical 36.6-kDa protein | Signal | 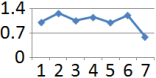 |
| S190 | G066P69 | At3g04810 | NIMA-related protein kinase | Signal | 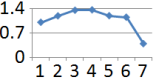 |
| S161 | N020F05 | At4g16830 | Nuclear RNA-binding protein A | Signal | 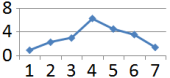 |
| S025 | B007P13.5pR | At2g28790 | Osmotin-like protein precursor | Signal | 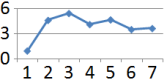 |
| S124 | G125P38 | At1g78300 | Possibly putative tyrosine activation protein | Signal | 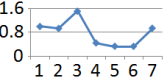 |
| S073 | T047H02 | At2g28790 | Possibly thaumatin-like protein | Signal | 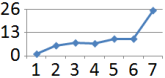 |
| S196 | N016D06 | At1g02320 | Protein-tyrosine kinase HTK156 | Signal | 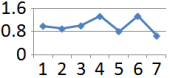 |
| S192 | G078P61.3pR | At5g56040 | Receptor protein kinase | Signal | 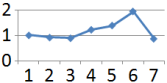 |
| S134 | UB11CPD04.3pR | At5g55190 | Small GTP-binding protein Ran1 homolog | Signal | 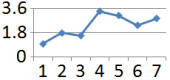 |
| **S184** | **P074A11** | **At2g29500** | **18.5-kDa class I heat shock protein** | **Transcription factor** | **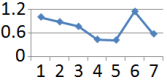** |
| S188 | P019F02 | At5g59720 | 18.5-kDa class I heat shock protein | Transcription factor | 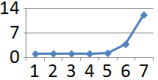 |
| S174 | P082E06 | At4g10250 | 22.0-kDa class IV heat shock protein precursor | Transcription factor | 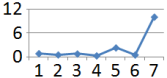 |
| S089 | G133P73 | No hit | B1148D12.15 protein | Transcription factor | 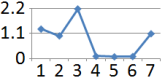 |
| S006 | X035D09 | At3g21890 | Calmodulin zinc finger protein, putative | Transcription factor | 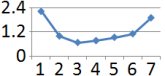 |
| S011 | T073A07 | At2g32070 | CCR4-associated factor protein | Transcription factor | 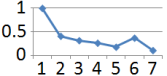 |
| S077 | A034P52.3pR | At5g10770 | CND41-like protein | Transcription factor | 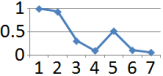 |
| S002 | R064H11 | No hit | Myb-related transcription factor | Transcription factor | 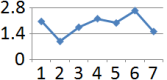 |
| S112 | R064H11 | No hit | Myb-related transcription factor | Transcription factor | 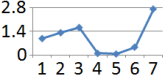 |
| S129 | R064H11 | No hit | Myb-related transcription factor | Transcription factor | 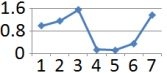 |
| S111 | R064H11 | No hit | Myb-related transcription factor, putative | Transcription factor | 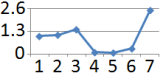 |
| **S022** | **P076C10** | **At1g75500** | **Nodulin-like protein** | **Transcription factor** | **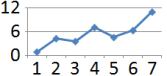** |
| **S035** | **A012P31.3pR** | **At1g75500** | **Nodulin-like protein** | **Transcription factor** | **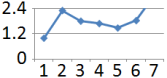** |
| S003 | T001C08 | At5g43810 | PINHEAD | Transcription factor | 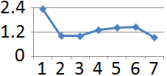 |
| **S069** | **G123P62.3pR** | **At1g23740** | **Putative auxin-induced protein** | **Transcription factor** | **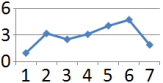** |
| **S010** | **S012A05** | **At4g39550** | **Related to transcription factor KCS1.** | **Transcription factor** | **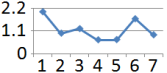** |
| S137 | PTRI29.5pR | At3g45260 | Zinc finger-like protein | Transcription factor | 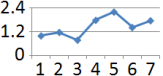 |
| S050 | P038E09 | At1g62480 | Ag13 protein precursor | Unknown | 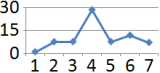 |
| S201 | A078P36.5pR | At4g12640 | Anther-specific S18 Protein | Unknown | 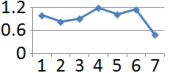 |
| **S061** | **UB62BPG02** | **At3g62140** | **P0510C12.9 protein** | **Unknown** | **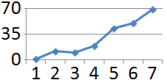** |
| S043 | R046A12 | At4g08950 | Phi-1-like phosphate-induced protein | Unknown | 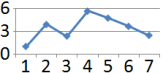 |
| **S055** | **UB56BPH03.5pR** | **At5g19875** | **Expressed protein** | **Unknown** | **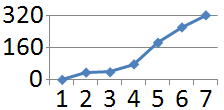** |
| S065 | A039P43.3pR | At2g12420 | Phloem-specific protein | Unknown | 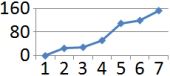 |
| S030 | B004P30.3pR | At4g02450 | Ripening-regulated protein DDTFR8 | Unknown | 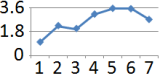 |
| **S083** | **UA43BPD01** | **At3g17210** | **Stable protein A** | **Unknown** | **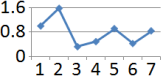** |

* Transcriptional pattern charts are based on cDNA microarray data. The *x*-axis represents different regeneration stages (from 1 to 7, representing 6, 10, 12, 14, 16, 18, and 22 days AG, respectively); the *y*-axis shows the ratio of the normalized data (intensity) of each stage to that at 6 days AG. Ten genes whose transcriptional profiles were confirmed by real-time PCR are shown in bold.
