# Supplementary material for: Dynamic changes in transcripts during regeneration of the secondary vascular system in Populus tomentosa Carr. revealed by cDNA microarrays
Source: BMC Genomics. 2009 May 11;10:215. doi: 10.1186/1471-2164-10-215 (PMC2685409; doi:10.1186/1471-2164-10-215)
Supplement: Additional file 4 — Sequences of primers used in real time RT-PCR. Sequences of primers used in real time RT-PCR. [file 1471-2164-10-215-S4.doc]

| **Name** | **Primer sequences (forward/reverse)** |
| --- | --- |
| S010 | GTGTCCAATGCGGCACAAC / TGATCCTCCGACCCTTCATG |
| S022 | CGGCCGCTCGAAATCC / AAGGCGATTAAGTTGGGTAACG |
| S035 | GGCAAAAGTGAGGAAAAGAAGTTC / TGCCGGTCAAACATTTTCTG |
| S055 | GCACATGGATGGGTGAGAGA / GGGACCAGTAAACCAGCAGCTA |
| S061 | TCACTGGCCGTCGTTTTACA / ATTCAGGCTGCGCAACTGTT |
| S069 | GGTCATTTATCCGATCCCATGA / TCCACCAGTATCCCCCATACA |
| S083 | GTGCCCCAATTGAAACTCTTCA / CATGGCAACCAGAACTCCAA |
| S175 | GCAAAAACTACCGACACAGCAA / CATGGCGTTGTAGGAGGCTAA |
| S184 | TCGCCCTATAGTGAGTCGTATTACA / ATGTGCTGCAAGGCGATTAAGT |
| S185 | TCACTGGCCGTCGTTTTACA / ATTCAGGCTGCGCAACTGTT |
| UBQ^**^ | GTTGATTTTTGCTGGGAAGC / GATCTTGGCCTTCACGTTGT |

Additional file 4: primer sequences for real time PCR^*^

^*^Primers are designed using Primer Express 3.0 (AppliedBiosystems)

^**^The primer sequences of the endogenous control UBQ was directly selected from Brunner et al. 2004.
